# Supplementary material for: MetaRibo-Seq measures translation in microbiomes
Source: Nat Commun. 2020 Jun 29;11:3268. doi: 10.1038/s41467-020-17081-z (PMC7324362; doi:10.1038/s41467-020-17081-z)
Supplement: Supplementary file 10 — Supplementary Data 7 [file 41467_2020_17081_MOESM10_ESM.zip › File2/Confidence_VeryHigh_Taxonomy/278392_out.krona.html]

Javascript must be enabled to view this page.

members
magnitude
magnitudeUnassigned
count
unassigned
taxon
rank

278392\_out

8

8
superkingdom
2

8
phylum
1239

186801
class
8

186802
order
8

family
541000
7

7
1263
genus

7

SRS015264\_contig\_number\_contig-100\_30425.30425SRS015578\_contig\_number\_contig-100\_3906.206842SRS051610\_contig\_number\_3158SRS053335\_contig\_number\_18540SRS075821\_contig\_number\_contig-100\_5737.144977SRS098571\_contig\_number\_68857SRS104197\_contig\_number\_contig-100\_703.6862
species
46228

1
31979
family

1
1649459
genus

154046
species

SRS043768\_contig\_number\_contig-100\_18570.18571
1
